# Type 2 Diabetes and Cardiovascular Factors Contrasted with Fibrinolysis Disorders in the Blood of Patients with Peripheral Arterial Disease

**DOI:** 10.3390/medicina55070395

**Published:** 2019-07-22

**Authors:** Radosław Wieczór, Anna Maria Wieczór, Arleta Kulwas, Danuta Rość

**Affiliations:** 1 Department of Pathophysiology, Faculty of Pharmacy, Nicolaus Copernicus University in Toruń, Ludwik Rydygier Collegium Medicum in Bydgoszcz, 85-094 Bydgoszcz, Poland; 2 Clinic of Vascular and Internal Medicine, Dr Jan Biziel University Hospital No. 2 in Bydgoszcz, 85-168 Bydgoszcz, Poland

**Keywords:** type 2 diabetes, peripheral arterial disease, fibrinolysis, cardiovascular risk factors

## Abstract

*Background and objectives:* Both in the pathogenesis of type 2 diabetes (DM 2) and Peripheral Arterial Disease (PAD), a vital role is played by endothelial dysfunction. Metabolic disorders found in DM 2 (hyperglycemia, insulin resistance), endothelial dysfunction, and increased inflammation lead to intensified atherothrombosis. The fibrinolysis system comprises a natural compensatory mechanism in case of hypercoagulability. The aim of this study was to assess concentrations of selected fibrinolysis parameters in the blood of patients with symptomatic PAD, including in particular concurrent DM 2 and other cardiovascular factors. *Materials and Methods:* In the group of 80 patients with PAD (27 F/53 M) and 30 healthy volunteers (10 F/20 M), the following parameters were measured: Concentrations of fibrinogen, tissue-Plasminogen Activator (t-PA Ag), Plasminogen Activator Inhibitor-1 (PAI-1 Ag), D-dimer, and platelet (PLT) count. *Results:* In the blood of patients with PAD and concomitant DM 2 significantly higher concentrations of fibrinogen were found in comparison with patients with PAD and without diabetes (*p* = 0.044). No significant impact was observed in individuals with atherosclerotic complications (manifested by coronary artery disease, atherosclerosis of cerebral arteries) and selected cardiovascular risk factors (smoking, LDL and triglyceride concentrations, BP values) on the levels of t-PA, PAI-1, D-dimer, and PLT count. It was found that t-PA Ag and PAI-1 Ag values tended to rise along with a BMI increase in the subgroups of subjects (with normal body mass, overweight, and obesity), but no statistically significant differences were observed. However, two significant positive correlations were reported between t-PA Ag and BMI, as well as between PAI-1 Ag and BMI. *Conclusions:* Type 2 diabetes in peripheral arterial disease affects the concentration of fibrinogen causing its increase, which is connected with the inflammation and prothrombotic process in the course of both conditions. The concurrence of atherosclerosis of coronary or cerebral arteries, smoking, LDL and TG concentrations, and BP value do not have a significant impact on the levels of analyzed fibrinolysis parameters. A positive correlation between BMI and t-PA Ag and PAI-1 Ag concentrations needs to be supported in further studies on a larger number of overweight and obese patients.

## 1. Introduction

Peripheral Arterial Disease (PAD) is a serious clinical and social problem mainly due to the growing number of patients, and complications that often lead to amputation and death. More than three hundred atherosclerosis risk factors are currently known, of which more than a dozen are significant in daily clinical practice. The causes of atherosclerosis can be divided into unmodifiable and modifiable causes, and the latter ones are of the utmost clinical importance in terms of prophylaxis and therapy possibilities [1,2]. Individual stratification of cardiovascular risk, multi-directional prophylaxis, and elimination of cardiovascular risk factors are highly important [3].

Unmodifiable factors include age (over 55 years for women and over 45 years for men), a positive family history of atherosclerosis—that is early, in men under 55 and in women under 65, family history of coronary artery disease or other artery diseases associated with atherosclerosis, and already diagnosed artery disease associated with atherosclerosis.

Key modifiable factors consist of smoking; the so-called ‘sedentary lifestyle’, namely, low physical activity; incorrect diet (including, but not limited to, excess of high-saturated fat foods, B-group vitamins, and folic acid deficiencies); lipid disorders including elevated levels of cholesterol—low-density lipoprotein (LDL), reduced concentration of high density lipoprotein (HDL), and increased triglyceride concentrations; hypertension, disorders of carbohydrate metabolism, i.e., impaired glucose tolerance (prediabetes) or diabetes; being overweight (according to Body Mass Index—BMI = 25–29.9 kg/m^2^); or obesity (BMI ≥ 30 kg/m^2^). In addition, elevated levels of C-Reactive Protein (CRP), homocysteine, lipoprotein (a), and fibrinogen can be observed in the blood of patients with atherosclerosis [1,2]. A high concentration of fibrinogen is an independent cardiovascular risk factor. Fibrinogen exerts an adverse impact on vascular walls, increases their permeability as well as chemotaxis and proliferation of smooth muscle cells, monocytes, leukocytes, and fibroblasts [4]. Metabolic disorders in type 2 diabetes (DM 2), such as hyperglycemia and insulin resistance, lead to intensified atherothrombosis because of endothelial dysfunction and increased inflammation [5].

Both in the pathogenesis of DM 2 and PAD, a vital role is played by endothelial dysfunction [6]. Vascular endothelium is involved in the formation of atherosclerotic plaque responsible for limb ischemia in PAD. In the place of endothelial damage, blood coagulation and micro-thrombosis are activated. The condition of the fibrinolysis system determines their lysis due to plasmin generated by plasminogen. Studies on the fibrinolysis system in the blood of patients with PAD have been the subject of only a few publications so far, and conclusions from the available studies indicate the complex impact of numerous different clinical and biochemical factors on the fibrinolytic potential in these patients [7]. However, the role of DM in PAD is not completely understood, and the exact prevalence of PAD in people with DM has been difficult to determine [8].

The aim of the study was to assess concentrations of selected fibrinolysis parameters in the blood of patients with symptomatic PAD, including in particular concomitant DM 2 and other cardiovascular factors.

## 2. Materials and Methods

### 2.1. Study Population

The study group included 80 patients with PAD (27 women and 53 men), with an average age of 63.5 ± 9 years. The symptomatic PAD was defined as a positive history of symptoms and a physical exam (intermittent claudication, decreased or absent pulses, muscle atrophy, ulcers), and resting ankle-brachial index (ABI) ≤ 0.9. Toe-brachial index (TBI) was not performed, but all patients had Doppler ultrasound evaluation (extent of obstruction). All study procedures were performed before angiography or angioplasty. DM 2 was defined as a positive history of this disease irrespective of the treatment method (diet, oral medication, insulin). Coronary artery disease (CAD) was defined as a positive history of stable ischemic heart disease, myocardial infarction (more than six months ago), coronary angioplasty, or coronary artery bypass grafting (CABG). Cerebral artery disease (CerAD) was defined as a positive history of carotid atherosclerosis (confirmed, e.g., by ultrasound of carotid arteries: Artery stenosis, thickness of the Intima-Media complex), stroke more than half a year ago, or Transient Ischemic Attack (TIA). The control group consisted of 30 healthy non-smoking sex-matched volunteers (10 women and 20 men), with an average age of 56 ± 6 years. The exclusion criteria included the lack of consent to participate in the research, the history of cancer, acute cardiovascular incident during the last six months (myocardial infarction, unstable coronary disease, PTCA, CABG, stroke, or TIA), diabetic retinopathy, diabetic neuropathy (in basic tests: Semmes Weinstein Monofilament, 128 Hz pitch pipe/tuning fork, neurotips) and severe Diabetic Foot Syndrome (with surgical intervention in last month), a surgical operation during last month, history of venous thromboembolism, use of anticoagulants (acenocoumarol, warfarin, dabigatran, rivaroxaban, apixaban), nephrotic syndrome, systemic connective tissue diseases, asthma, chronic obstructive pulmonary disease, inflammatory bowel disease, and pregnant women. The studies were authorized by the local Bioethics Commission of Ludwik Rydygier Collegium Medicum in Bydgoszcz, Nicolaus Copernicus University in Toruń, no. KB 509/2011 (approved on 11 October 2011) and were carried out in accordance with the Declaration of Helsinki. Participation in the research was voluntary and preceded by obtaining informed consent.

### 2.2. Laboratory Analysis

The research material was 15 mL of venous blood sampled in the morning hours after a night’s rest. The obtained material was used to determine the concentrations of fibrinogen by means of CC-3003 apparatus (Bio-ksel®, Grudziądz, Poland), concentrations of tissue-Plasminogen Activator (t-PA Ag, Diagnostica Stago®, Asnières-sur-Seine, France) and Plasminogen Activator Inhibitor-1 (PAI-1 Ag, Sekisui Diagnostics®, USA) using the immunoenzymatic method (ELISA), D-dimer concentrations using CC-3003 apparatus (Bio-ksel®, Grudziądz, Poland), and platelet (PLT) count by means of XT-4000i apparatus (Symex®, Hyogo, Japan).

### 2.3. Statistical Analysis

The statistical analysis was conducted using Statistica 12.0 software (StatSoft®, Cracow, Poland). The compatibility of examined parameters distribution with the standard normal distribution was assessed by the W Shapiro–Wilk test. The significance level was set at *p* < 0.05.

## 3. Results

Table 1 presents the overall characteristics of the test group.

Men were predominant in the study group, mainly patients in Rutherford category 1–3 (81%), with DM 2 in ca. one-third of cases. Yang et al. reported that the exact prevalence of PAD in patients with diabetes has been difficult to determine because of many factors, including the absence of specific symptoms, unsettled pain perception due to peripheral neuropathy, and inadequate screening [8]. In this study 45% of subjects were reported to have concurrent CAD, whereas according to reference literature this incidence is estimated to be 10–90% [6,9]. In one out of four subjects a positive history of CerAD was found. Abnormal BMI characterized over 60% of the subjects, and the vast majority of them were with smoking and hypertension load. Despite a relatively small number of subjects, considering the epidemiological data from large observational trials, this group can be regarded as representative.

Table 2 displays values of tested parameters depending on whether DM 2 is coexisting or not.

In the blood of patients with PAD and concurrent DM 2 or without DM 2, significantly higher concentrations of fibrinogen, t-PA Ag, PAI-1 Ag, and D-dimer were reported against the control group. Significantly higher levels of fibrinogen characterized subjects with PAD and coexisting DM 2 as compared to those with PAD and without DM 2 (*p* = 0.044).

Table 3 shows a comparison of measured parameters in the subgroups of patients with PAD and concurrent CAD, without concurrent ischemic heart disease, and in the control group.

Significantly elevated levels of fibrinogen, t-PA Ag, PAI-1 Ag, and D-dimer were observed in patients with or without concurrent coronary artery disease when compared to the control group. No significant differences were noticed between subgroups separated from the test group on the basis of the medical history of coexisting CAD.

Table 4 provides a comparison of the values of studied parameters in the subgroups of patients with concurrent CerAD, without CerAD, and in the control group.

Significantly higher values of fibrinogen, t-PA Ag, PAI-1 Ag, and D-dimer were reported in both studied subgroups as compared to the control group. Between the values of analyzed parameters obtained in the subgroups of patients with PAD and with or without coexisting CerAD, no significant differences were seen.

Table 5 summarizes the levels of studied parameters depending on BMI in patients with PAD and in the control group.

Statistically significant differences were observed between the values of parameters in patients in the subgroups according to BMI when compared to the control group. No distinctive differences were obtained between subgroups of patients with PAD divided on the basis of BMI into subgroups with normal body weight, overweight, or obesity, but it was observed that the concentrations of t-PA Ag and PAI-1 Ag tended to increase in these subgroups while the D-dimer concentrations decreased.

Table 6 compares the values of analyzed parameters in the test group divided into three subgroups: The overall number of smokers—among them the number of current smokers or former smokers, non-smokers, and in the control group.

It was found that the concentration of fibrinogen was significantly elevated in current and former smokers compared to the control group. The concentrations of t-PA Ag, PAI-1 Ag, and D-dimer were significantly higher among individuals with a medical condition regardless of smoking (currently or in the past) or non-smoking as compared to the control group. No distinctive differences were obtained between the values of studied parameters in the subgroups categorized according to the smoking status.

Table 7 summarizes the results of the analysis of correlations between the studied parameters (concentrations of fibrinogen, t-PA Ag, PAI-1 Ag, D-dimer, PLT count) and selected clinical parameters such as the number of pack-years, BMI values, levels of LDL, triglycerides (TG), and blood pressure (BP) values in the test group taking into account statistically significant results.

The analysis of studied biochemical and clinical parameters conducted in the PAD group only revealed statistically significant positive correlations of body mass index values and the concentrations of t-PA Ag and PAI-1 Ag.

## 4. Discussion

This research did not indicate any significant influence of coronary artery disease, atherosclerosis of cerebral arteries, overweight, obesity, smoking, LDL, and triglycerides concentrations on the concentration of fibrinogen in patients with PAD.

These studies have shown, however, that fibrinogen concentration is most affected by concomitant type 2 diabetes. The analysis on subgroups of patients with PAD and concurrent type 2 diabetes (PAD-DM2+, *n* = 28) and without diabetes (PAD-DM2-, n = 52) demonstrated statistically significant differences, with a clear advantage of fibrinogen concentration in the blood of patients with PAD and concomitant type 2 diabetes. The study conducted by Zalewska-Rydzkowska et al. did not show any distinctive differences between the group of patients with peripheral arterial disease and the subgroup with type 2 diabetes in terms of fibrinogen concentration [10]. The lack of significant differences between patients with type 2 diabetes and coexisting PAD and diabetic patients without PAD in terms of fibrinogen concentrations was reported by Hutajulu et al. [11]. Gosk-Bierska et al. did not observe any differences between fibrinogen concentrations in groups of patients with PAD and diabetes and patients with PAD and without diabetes, but these values were significantly higher in both groups as compared to the control group [12]. It can be considered that hyperglycemia coexisting with PAD in type 2 diabetes influences fibrinogen concentrations, causing their increase. Fibrinogen as an acute-phase protein indicates inflammation in the course of atherosclerosis and diabetes, and is a thrombosis risk factor.

This research study did not indicate a significant impact of certain atherosclerosis complications (including coronary artery disease, atherosclerosis of cerebral arteries) and selected cardiovascular risk factors (smoking, LDL, and triglyceride concentrations) on the levels of t-PA and PAI-1. It was found that t-PA Ag and PAI-1 Ag values tended to rise along with BMI increase in the subgroups of subjects (with normal body mass, overweight, and obesity), though without statistically significant differences. However, significant positive correlations were found between t-PA Ag and BMI as well as between PAI-1 Ag and BMI. The analysis of available literature has shown that positive correlations of PAI-1 Ag and BMI concentrations were observed in patients after myocardial infarction and individuals with morbid obesity [13,14]. Alessi et al. explain this observation by the fact that adipose tissue in abdominal obesity is where synthesis of PAI-1 occurs [14]. No data were found about a positive correlation of t-PA Ag and BMI in the blood of both sick and healthy subjects. Indirect data, however, seem to suggest that between the concentration of t-PA Ag and BMI there is a negative correlation. This is supported by the research conducted by Abd El-Kader and Al-Jiffri, who evidenced that a low BMI in patients with simple obesity increased the levels of t-PA Ag and t-PA activity [15]. It is possible that in our research study a positive correlation of the concentration of t-PA Ag and BMI as well as PAI-1 Ag and BMI was associated with high plasma levels of PAI-1 Ag and reflects intensive formation of t-PA-PAI-1 complexes.

In the study by Kotschy et al., concentrations of t-PA and PAI-1 were determined in patients with PAD and concomitant diabetes and in patients with PAD and without diabetes, and no significant differences were observed in the levels of these endothelial markers between the groups and in comparison with the control group. The authors of the quoted research study indicated the common etiology of type 2 diabetes and PAD manifested by endothelial dysfunction of blood vessels [16]. Likewise, Hutajulu et al. observed no distinctive differences between patients with type 2 diabetes and concomitant PAD and patients with diabetes and without PAD in terms of PAI-1 concentrations [11]. This research study did not demonstrate the influence of concurrent type 2 diabetes on t-PA Ag and PAI-1 Ag concentrations in PAD patients.

This study indicated significantly higher D-dimer levels in the blood of patients with symptomatic PAD as compared to healthy subjects, but it did not evidence a distinctive impact of selected concurrent diseases (coronary artery disease, atherosclerosis of cerebral arteries) and certain selected cardiovascular risk factors (type 2 diabetes, overweight, obesity, smoking, LDL, and triglycerides concentrations) on the levels of this parameter. A comparative analysis of two groups of patients in the publication by Zalewska-Rydzkowska et al. demonstrated significantly higher levels of fibrin degradation products (FDP) in the blood of patients with peripheral obstructive artery disease as compared to subjects with type 2 diabetes [10].

This research study used a laboratory parameter easily accessible in everyday clinical practice, that is, the absolute value of PLT. However, this study did not show a significant impact of coronary artery disease coexisting with PAD on platelet count, and no distinctive influence of concomitant atherosclerosis of cerebral arteries was found. In most observations of type 2 diabetes, lower PLT levels are reported which can be explained by shortened platelet survival due to hyperglycemia and oxidation stress, increased rotation, and/or impaired production in bone marrow [17,18,19,20]. In our research, patients with PAD and concomitant type 2 diabetes were not found to have any differences in PLT count as compared to subjects with peripheral arterial disease without diabetes. The study conducted by Gosk-Bierska et al. comparing groups of patients with PAD and concurrent type 2 diabetes and the group of patients with PAD and without diabetes did not display any significant differences in PLT levels [12]. Similar findings including no difference between PLT levels in diabetic patients with PAD against non-diabetic patients with PAD were obtained by Li et al. They found significantly the highest mean platelet volume (MPV) ratio in patients with diabetes and PAD [21]. An interesting application of a platelet count appears to be the so-called platelet-to-lymphocyte ratio (PLR). PLR was investigated by Gary et al. who demonstrated in their retrospective study on large test group (*n* = 2121) that critical limb ischemia significantly more frequently occurred in patients with PLR > 150. Moreover, this group represented more cases of myocardial infarction [22].

This research study did not show an impact of other factors such as BMI, smoking or concentrations of cholesterol, LDL, and triglycerides on PLT count in the blood of patients suffering from peripheral arterial disease.

## 5. Conclusions

Type 2 diabetes in peripheral arterial disease affects fibrinogen concentration causing its increase, which is connected with inflammation and the prothrombotic process in the course of both of these conditions.

The concurrence of atherosclerosis of coronary or cerebral arteries, smoking, LDL, and TG concentrations and blood pressure value do not have a significant impact on the concentrations of analyzed fibrinolysis parameters.

Positive correlations observed between BMI and t-PA Ag levels and concentrations of PAI-1 Ag and BMI can be associated with high plasma levels of PAI-1 Ag, and manifest an intensive formation of t-PA-PAI-1 complexes, which needs to be supported in further studies on a larger number of overweight or obese patients with PAD.

## Figures and Tables

**Table 1:** Patient demographics and risk factors.

Parameter	Unit	Value
Sex (women/men)	n (%)/n (%)	27 (34%)/53 (66%)
Average age ± SD	years	63.5 ± 9
Number of patients in Rutherford category 1–3	n (%)	65 (81%)
Number of patients in Rutherford category 4	n (%)	4 (5%)
Number of patients in Rutherford category 5–6	n (%)	11 (14%)
Number of patients with DM 2	n (%)	28 (35%)
Mean HbA1c in patients with DM 2 ± SD	%	7.9 ± 1.1
Number of patients with CAD	n (%)	36 (45%)
Number of patients with CerAD	n (%)	19 (24%)
Number of patients with HA	n (%)	71 (89%)
Number of overweight patients	n (%)	32 (40%)
Number of obese patients	n (%)	18 (22.5%)
Mean BMI ± SD	kg/m^2^	26.4 ± 4.4
Total number of smokers (current and former)	n (%)	74 (92.5%)
Number of current smokers	n (%)	27 (34%)
Average number of pack-years ± SD	n	32.7 ± 18.8
Number of non-smokers	n (%)	6 (7.5%)
Mean LDL ± SD	mg/dL	119.7 ± 39.3
Mean TG ± SD	mg/dL	143.9 ± 73.3

Diabetes type 2 (DM 2); Coronary artery disease (CAD); Cerebral artery disease (CerAD).

**Table 2:** The values of analyzed parameters in the test group in the subgroups with concurrent type 2 diabetes (DM2+) and without diabetes (DM2-) and in the control group (without diabetes).

Analyzed Parameter and Unit	Assumed Value	Test Group (PAD, *n* = 80)		Control Group (C, *n* = 30) *c*	*p*
		DM2+ (*n* = 28) *a*	DM2− (*n* = 52) *b*		
Fibrinogen (g/L)	Me (Q25; Q75)	5.16 (3.9; 5.81)	4.21 (3.6; 4.84)	3.36 (2.8; 3.7)	**a vs. b = 0.044** a vs. c < 0.001 b vs. c < 0.001
t-PA Ag (ng/mL)	Me (Q25; Q75)	13.37 (10.97; 16.5)	11.62 (8.57; 16.23)	4.79 (2.62; 5.77)	a vs. b NS a vs. c < 0.001 b vs. c < 0.001
PAI-1 Ag (ng/mL)	X (±SD)	48.14 (±14.03)	48.7 (±14.19)	17.79 (±6.91)	a vs. b NS a vs. c < 0.001 b vs. c < 0.001
D-dimer (ng/mL)	X (±SD)	874.76 (±253.45)	807.67 (±284.72)	312.58 (±93.25)	a vs. b NS a vs. c < 0.001 b vs. c < 0.001
PLT (G/L)	Me (Q25; Q75)	244.5 (202; 316.5)	246.5 (207.5; 282.5)	223 (182; 282)	a vs. b NS a vs. c NS b vs. c NS

Me = median, Q25 = lower quartile, Q75 = upper quartile, X = mean, SD = standard deviation. Bold data represent the most important significant difference.

**Table 3:** Values of analyzed parameters in the test group, in the subgroups of patients with concurrent coronary artery disease (CAD+) and without it (CAD-) and in the control group (without artery disease).

Analyzed Parameter and Unit	Assumed Value	Test Group (PAD, *n* = 80)		Control Group (C, *n* = 30) *c*	*p*
		CAD+ (*n* = 36) *a*	CAD− (*n* = 44) *b*		
Fibrinogen (g/L)	Me (Q25; Q75)	4.34 (3.52; 5.13)	4.55 (3.69; 5.22)	3.36 (2.8;3.7)	a vs. b NS a vs. c < 0.001 b vs. c < 0.001
t-PA Ag (ng/mL)	Me (Q25; Q75)	13.52 (10.35; 17.76)	11.62 (8.69; 14.26)	4.79 (2.62;5.77)	a vs. b NS a vs. c < 0.001 b vs. c < 0.001
PAI-1 Ag (ng/mL)	X (±SD)	48.16 (±15.81)	48.77 (±12.61)	17.79 (±6.91)	a vs. b NS a vs. c < 0.001 b vs. c < 0.001
D-dimer (ng/mL)	X (±SD)	754.7 (±277.77)	892.77 (±260.02)	312.58 (±93.25)	a vs. b NS (0.056) a vs. c < 0.001 b vs. c < 0.001
PLT (G/L)	Me (Q25; Q75)	240 (206; 266.5)	254.5 (202; 303)	223 (182; 282)	a vs. b NS a vs. c NS b vs. c NS

Me = median, Q25 = lower quartile, Q75 = upper quartile, X = mean, SD = standard deviation.

**Table 4:** The values of studied parameters in the group studied in the subgroups of patients with concurrent cerebral artery disease (CerAD+) and without it (CerAD-), and in the control group (without cerebral artery disease).

Analyzed Parameter and Unit	Assumed Value	Test Group (PAD, *n* = 80)		Control Group (C, *n* = 30) *c*	*p*
		CerAD+ (*n* = 19) *a*	CerAD− (*n* = 61) *b*		
Fibrinogen (g/L)	Me (Q25; Q75)	4.48 (3.81; 5.06)	4.53 (3.65; 5.27)	3.36 (2.8; 3.7)	a vs. b NS a vs. c < 0.001 b vs. c < 0.001
t-PA Ag (ng/mL)	Me (Q25; Q75)	13.33 (11.26; 16.36)	11.86 (8.61; 16.42)	4.79 (2.62; 5.77)	a vs. b NS a vs. c < 0.001 b vs. c < 0.001
PAI-1 Ag (ng/mL)	X (±SD)	50.92 (±7.06)	47.77 (±15.52)	17.79 (±6.91)	a vs. b NS a vs. c < 0.001 b vs. c < 0.001
D-dimer (ng/mL)	X (±SD)	882.05 (±284.08)	808.1 (±272.1)	312.58 (±93.25)	a vs. b NS a vs. c < 0.001 b vs. c < 0.001
PLT (G/L)	Me (Q25; Q75)	251 (196; 267)	244 (208; 296)	223 (182; 282)	a vs. b NS a vs. c NS b vs. c NS

Me = median, Q25 = lower quartile, Q75 = upper quartile, X = mean, SD = standard deviation.

**Table 5:** The values of studied parameters in the group studied in the subgroups according to the body mass index: With normal BMI, overweight, and obesity against the control group (with normal BMI).

Analyzed Parameter and Unit	Assumed Value	Test Group (PAD, *n* = 78) *			Control Group (C, *n* = 30) BMI = 18.5–24.9 *d*	*p*
		Normal BMI = 18.5–24.9 (*n* = 28) *a*	Overweight BMI = 25–29.9 (*n* = 32) *b*	Obesity BMI ≥ 30 (*n* = 18) *c*		
Fibrinogen (g/L)	Me (Q25; Q75)	4.15 (3.69; 4.96)	4.67 (3.52; 5.15)	4.26 (3.76; 5.33)	3.36 (2.8; 3.7)	a vs. b vs. c NS a vs. d < 0.001 b vs. d < 0.001 c vs. d < 0.001
t-PA Ag (ng/mL)	Me (Q25; Q75)	11.25 (7.29; 15.73)	12.55 (9.61; 15.36)	14.73 (11.18; 18.06)	4.79 (2.62; 5.77)	a vs. b vs c NS a vs. d < 0.001 b vs. d < 0.001 c vs. d < 0.001
PAI-1 Ag (ng/mL)	Me (Q25; Q75)	44.85 (28.1; 55.63)	51 (44,8; 55,91)	52.7 (45,29; 61,58)	16.81 (14.02; 22.01)	a vs. b vs. c NS a vs. d < 0.001 b vs. d < 0.001 c vs. d < 0.001
D-dimer (ng/mL)	X (±SD)	871.54 (±266.25)	809.2 (±263.14)	784.98 (±325.59)	312.58 (±93.25)	a vs. b vs. c NS a vs. d < 0.001 b vs. d < 0.001 c vs. d < 0.001
PLT (G/L)	Me (Q25; Q75)	266 (207; 315)	233.5 (201.5; 284.5)	236(208; 265)	223 (182; 282)	a vs. b vs. c NS a vs. d NS b vs. d NS c vs. d NS

* 2 patients with BMI < 18.5 not taken into account in the analysis. Me = median, Q25 = lower quartile, Q75 = upper quartile, X = mean, SD = standard deviation.

**Table 6:** The values of studied parameters in the studied group within subgroups of: Smokers (current or former) and non-smokers against the control group (non-smokers).

Analyzed Parameter and Unit	Assumed Value	Test Group (PAD, *n* = 80)			Control Group (C, *n* = 30) *d*	*p*
		Smokers (*n* = 74)		Non-Smokers (*n* = 6) *c*		
		Current (*n* = 27) *a*	Former (*n* = 47) *b*			
Fibrinogen (g/L)	Me (Q25; Q75)	4.79 (3.54; 5.43)	4.2 (3.71; 4.99)	4.23 (2.78; 5.38)	3.36 (2.8; 3.7)	(a+b) vs. c NS a vs. c NS b vs. c NS a vs. b NS a vs. d < 0.001 b vs. d < 0.001 c vs. d NS (a+b) vs. d < 0.001
t-PA Ag (ng/mL)	Me (Q25; Q75)	13.12 (11.12; 15.8)	11.94 (8.61; 16.57)	12.28 (8.69; 16.42)	4.79 (2.62; 5.77)	(a+b) vs. c NS a vs. c NS b vs. c NS a vs. b NS a vs. d < 0.001 b vs. d < 0.001 c vs. d = 0.016 (a+b) vs. d < 0.001
PAI-1 Ag (ng/mL)	X (±SD)	43.42 (±15.19)	50.99 (±12.83)	48.39 (±16.19)	17.79 (±6.91)	(a+b) vs. c NS a vs. c NS b vs. c NS a vs. b NS (0.095) a vs. d < 0.001 b vs. d < 0.001 c vs. d < 0.001 (a+b) vs. d < 0.001
D-dimer (ng/mL)	X (±SD)	901.09 (±221.61)	788.06 (±284.76)	966.95 (±378.07)	312.58 (±93.25)	(a+b) vs. c NS a vs. c NS b vs. c NS a vs. b NS a vs. d < 0.001 b vs. d < 0.001 c vs. d < 0.001 (a+b) vs. d <0.001
PLT (G/L)	Me (Q25; Q75)	244 (203; 276)	249 (203; 290)	298 (238; 359)	223 (182; 282)	(a+b) vs. c NS a vs. c NS b vs. c NS a vs. b NS a vs. d NS b vs. d NS c vs. d NS (a+b) vs. d NS

Me = median, Q25 = lower quartile, Q75 = upper quartile, X = mean, SD = standard deviation.

**Table 7:** Analysis of correlations between the studied parameters.

PAD (*n* = 80)	Unit	Number of Pack–Years	BMI	LDL	TG	BP
		n	kg/m^2^	mg/dl	mg/dl	mmHg
Fibrinogen	g/L	NS	NS	NS	NS	NS
t-PA Ag	ng/mL	NS	*r =* 0.3 *p =* 0.037	NS	NS	NS
PAI-1 Ag	ng/mL	NS	*r* = 0.33 *p =* 0.019	NS	NS	NS
D-dimer	ng/mL	NS	NS	NS	NS	NS
PLT	G/L	NS	NS	NS	NS	NS
